# Chondroblastic Subtype Is Associated with Higher Rates of Local Recurrence in Skeletal Osteosarcoma

**DOI:** 10.3390/jcm14227952

**Published:** 2025-11-10

**Authors:** Alexandra N. Krez, Sarah Fagan-Kellogg, Laurie A. Graves, Aron Mebrahtu, Nicole L. Levine, Elizabeth Sachs, William C. Eward, Brian E. Brigman, Julia D. Visgauss

**Affiliations:** 1 Department of Orthopedic Surgery, Duke University Hospital, Durham, NC 27710, USA; aron.mebrahtu@duke.edu (A.M.);; 2 Department of Pediatrics, Duke University Hospital, Durham, NC 27710, USA; 3 Duke Cancer Institute, Duke University Hospital, Durham, NC 27710, USA

**Keywords:** osteosarcoma, local recurrence, chondroblastic

## Abstract

**Background/Objectives:** Locally recurrent osteosarcoma is associated with high patient morbidity and mortality, yet the risk factors for local recurrence remain incompletely understood. Therefore, the objective of this study was to comprehensively assess the impact of tumor histology, patient demographics, surgical resection, and chemotherapy delivery on the risk of local recurrence, development of metastases, and overall patient survival in osteosarcoma. **Methods:** This single-center retrospective review included 102 patients with skeletal osteosarcoma who underwent primary surgical resection between August 2001 and August 2021. Primary outcomes included the development and timing of local recurrence following primary resection. Data was abstracted from the electronic medical record, and statistical analyses were performed to identify demographic, tumor, or management (surgical and chemotherapy) related factors associated with an increased risk of local recurrence. **Results:** Locally recurrent osteosarcoma developed in 13.7% (*n* = 14) of patients after primary resection. Patients with local recurrence were more likely to have the chondroblastic subtype (57.1% versus 19.3% in those without local recurrence, *p* = 0.005). The chondroblastic subtype was associated with shorter local recurrence-free survival and overall survival than other histologic subtypes (*p* < 0.001). Resection approach, surgical margin status, histologically assessed response to neoadjuvant chemotherapy, and type and dose intensity of chemotherapy delivered were not statistically associated with risk of local recurrence. **Conclusions:** Chondroblastic histology is a risk factor for local recurrence in osteosarcoma and is associated with poor overall survival in our patient cohort.

## 1. Introduction

Osteosarcoma is the most common primary malignant bone tumor with an incidence of 3–4 per million people per year [1]. The management of osteosarcoma has evolved throughout the twentieth century with transition from predominantly limb amputations to a multi-modal approach including chemotherapy and limb salvaging resection. The principal objective of surgical management is to achieve optimal local tumor control while maximizing functional preservation. Despite advancements, the specter of locally recurrent osteosarcoma remains a devastating challenge, as it is associated with distant disease progression and mortality [2,3,4] with a 5-year overall survival rate of only approximately 30% [5,6].

Reported risk factors for the development of local recurrence (LR) include inadequate surgical margins [7,8,9], poor response to chemotherapy [10], pelvic tumor location [10,11] and delay in surgical management [12]. For example, rates of local recurrence associated with primary tumors in the pelvis ranges from 11 to 62% [8,13,14] in comparison to only 1–17% with primary tumors arising in the extremities [8,15,16,17]. However, our understanding of additional risk factors for LR in osteosarcoma remains incomplete, as many other treatment factors and aspects of tumor biology, such as histological subtype, may increase this risk.

The gap in our understanding of the tumor and treatment-specific risk factors for local recurrence represents a critical area of research need. Though there is significant heterogeneity in tumor biology among patients with osteosarcoma [18], we do not yet understand how to tailor treatment approaches for patients who are at the highest risk for local recurrence. This study aims to investigate the factors contributing to local recurrence and to evaluate their impact on survival outcomes. Specifically, it examines whether histologic subtype, particularly chondroblastic osteosarcoma, predicts local recurrence and survival. Furthermore, this study focuses on examining the differences in recurrence rates and survival outcomes between chondroblastic osteosarcoma and other osteosarcoma subtypes.

## 2. Materials and Methods

This was a single-center, retrospective review of adult and pediatric patients who underwent primary surgical resection for osteosarcoma between August 2001 and August 2021. The diagnosis of osteosarcoma was confirmed by histopathological examination. This study included patients with osteosarcoma of any grade, as well as those with metastatic disease at the time of diagnosis. However, patients with extra-skeletal osteosarcoma were excluded. Surgical resection encompassed limb sparing/wide surgical resection, rotationplasty, and amputation. All patients were evaluated either pre-operatively or post-operatively by a fellowship-trained orthopedic oncology surgeon at our tertiary academic medical center for the management of osteosarcoma. As the treatment of osteosarcoma is multidisciplinary, we did not exclude patients who received aspects of their care including chemotherapy or surgical resection at an outside hospital. Institutional review board approval for this study was provided prior to study initiation. This study was performed in accordance with the ethical standards in the 1964 Declaration of Helsinki and regulations of the US Health Insurance Portability and Accountability Act.

### 2.1. Clinical Data Collection

Data was manually extracted from patients’ electronic medical record. Demographic data obtained included age at primary resection, sex, race, body mass index (BMI) and smoking status. Surgical data collection included location of diagnostic biopsy (outside institution versus study institution) and resection procedure type (limb sparing, amputation or rotationplasty). Tumor characteristics were extracted from the operative pathology report including anatomic location and size, histologic subtype and grade, treatment response (percent necrosis), and margin status. Chemotherapy agents used in neoadjuvant and adjuvant systemic therapy, cumulative dose of chemotherapy received, and occurrence of deviations in chemotherapy delivery were recorded. Deviations included delay or unanticipated discontinuation of chemotherapy. Patients for whom records of chemotherapy delivery were not available (agents received and doses used) were excluded from the chemotherapy-related sub-analyses.

### 2.2. Outcomes

The primary outcomes of this study were the development and timing of LR following primary resection. Local recurrences were identified and confirmed by imaging as well as tissue pathology when available. Secondary outcome measures included metastasis-free and overall survival.

### 2.3. Statistical Analysis

Statistical analysis was performed with RStudio (R version 4.2.2). Demographic and clinical data were summarized for all subjects. Descriptive data are presented as mean ± standard deviation (SD) for continuous measures and count (% of total) for categorical variables. Bivariate analysis using χ^2^ test or Fisher’s exact test as appropriate for categorical variables was conducted to compare differences between patients who developed LR and those who do not. T tests or Wilcoxon Rank Sum Test were used to compare continuous data based on normality. Statistical analyses involving chemotherapy delivery was performed only on the subset of patients with intermediate or high-grade OS who received neoadjuvant and/or adjuvant chemotherapy. Multivariable logistic regression was performed to evaluate the association between chondroblastic histology and local recurrence, controlling for tumor grade, location, and surgical margins. *p* values < 0.05 were considered statistically significant. Time-to-event analyses for LR and overall survival were visualized with Kaplan–Meier analysis. Cox proportional hazards analysis was used to model association between histological subtype and time to LR and mortality.

## 3. Results

### 3.1. Patient Characteristics

One hundred and two patients underwent surgical resection for osteosarcoma and were included in this analysis. Patient demographics and differences amongst patient sex are summarized in Table 1 and Table 2. The mean age of the total cohort was 25.6 ± 16.9 years (range, 4–84 years), and 45.1% were female. Mean BMI was 25.2 ± 8.4 kg/m^2^. Most of the patients were non-smokers (78.4%).

### 3.2. Tumor Characteristics and Surgical Interventions

Tumors were most commonly located in the lower extremity (*n* = 76, 74.5%) followed by upper extremity (*n* = 18, 17.6%), pelvis (*n* = 4, 3.9%) and shoulder girdle (*n* = 4, 3.9%). The majority of the cohort underwent biopsy (85.3%) and surgical management (87.3%) at our institution. Surgical procedures included limb sparing/wide surgical resection (*n* = 87, 85%), amputation (*n* = 10, 10%) and rotationplasty (*n* = 5, 5%). Positive surgical margins were present in 11.8% (*n* = 12) of the total cohort. Of the 90 patients with negative surgical margins, the majority (68%) were noted to be within 1.0 cm. Of note, one patient with an above the knee amputation (AKA) had a positive soft tissue margin, and no patients with rotationplasty had a positive margin. The average largest dimension of the tumor was 11.0 ± 5.2 cm. Tumors in the majority of patients were classified as high grade on surgical pathology (86.3%). High-grade and intermediate histologic subtypes included osteoblastic (*n* = 45, 44.1%), chondroblastic (*n* = 25, 24.5%), telangiectatic (*n* = 10, 9.8%), fibroblastic (*n* = 4, 3.9%), high-grade surface (*n* = 2, 2.0%), small cell (*n* = 1, 1.0%), dedifferentiated (*n* = 1, 1.0%) and periosteal (*n* = 2, 2.0%) osteosarcoma. Low-grade subtypes included parosteal (*n* = 11, 10.8%) and low-grade central (*n* = 1, 1.0%) osteosarcoma (Table 2).

### 3.3. Chemotherapy Delivery

Of the ninety patients with intermediate or high-grade osteosarcoma, 69 patients (76.7%) received neoadjuvant chemotherapy. Seventy-five patients (83.3%) received adjuvant chemotherapy. A total of 80 patients received neoadjuvant and/or adjuvant chemotherapy. Tumors in approximately half of patients demonstrated ≥ 90% tumor necrosis in response to neoadjuvant therapy (*n* = 33; 51.6%). A list of chemotherapy agents received by patients is summarized in Table 3 and includes doxorubicin (*n* = 74, 92.5%), cisplatin (*n*= 74, 92.5%), high-dose methotrexate (*n* = 60, 75.0%), ifosfamide (*n* = 17, 20%) and etoposide (*n* = 14, 17.5%). Treatment disruptions/delays were experienced by more than half of the total cohort (63.8%). The median time to initiation of adjuvant chemotherapy after surgical resection was 29 [8–503] days.

### 3.4. Local Recurrence, Metastasis and Overall Patient Survival

**Figure 1:**
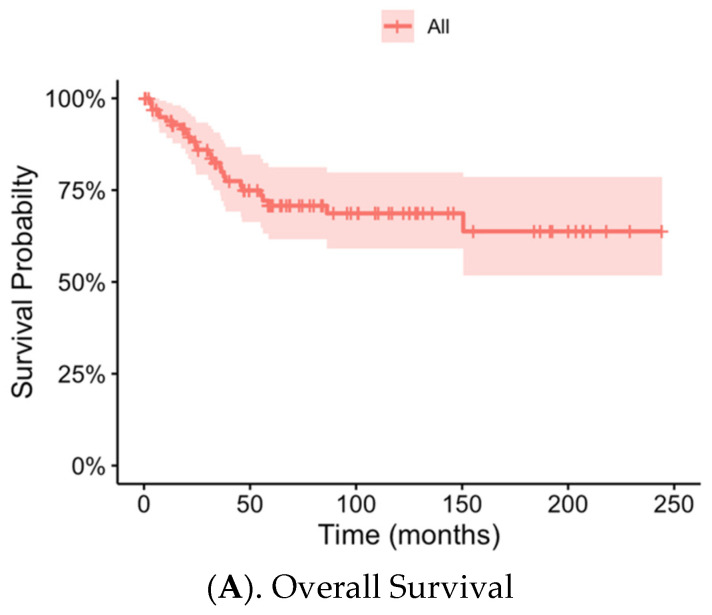

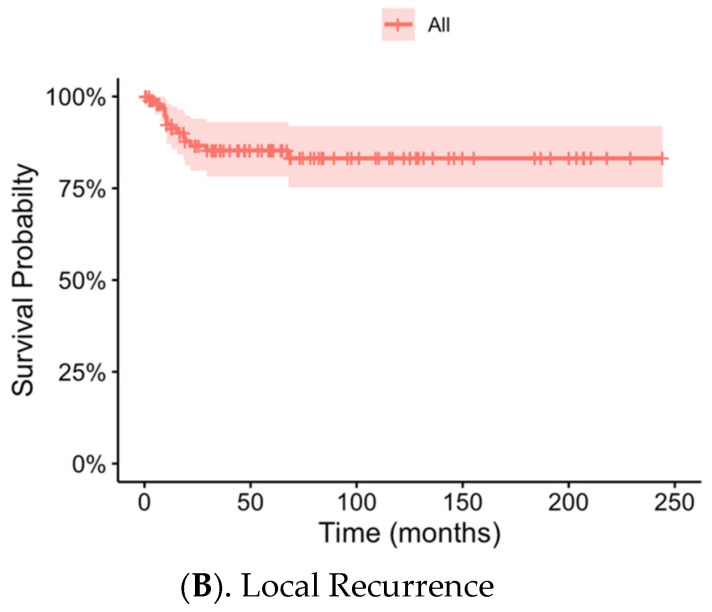
Kaplan–Meier curve for overall patient survival (**A**) and local recurrence (**B**) in patients with skeletal osteosarcoma.

The median follow-up duration was 60.7 months. Fourteen patients (13.7%) developed LR. LRs occurred more commonly in residual bone (n = 9) than soft tissues (n = 5). Five-year LR-free survival was 85.3% (95% CI [78.2–93.0%]) (Figure 1). Forty-eight patients (47.1%) developed metastatic disease at one or more distant sites, either at the time of diagnosis or during follow-up. Of these, 11 patients (9.8%) had metastatic disease at initial presentation. There was no significant difference in the proportion of patients with metastatic disease at diagnosis between those who developed local recurrence and those who did not (1/14 [7.1%] vs. 10/88 [11.4%]; *p* > 0.99). The most common location of metastasis was the lungs (n = 43). Seventy-five patients (73.5%) were alive at the time of data collection. The five-year overall survival was 70.7% (95% CI [61.6–81.2%]). The association between LR and distant metastatic disease was very high, with 86% of the 14 patients with LR also developing distant metastasis. However, when temporally reviewing the presentation of LR and metastasis in these patients, many developed their LR after (n = 4) or within 3 months (n = 5) of development of metastatic disease.

### 3.5. Risk Factors for Local Recurrence

There were no significant differences in age, sex, or BMI between patients who developed LR and those who did not (Table 1). However, there was a trend towards higher rates of prior or current tobacco use in patients who developed LR (35.7% vs. 19.3%, *p* = 0.18).

No significant differences in risk of LR were found with regard to specific chemotherapy agents received or response to chemotherapy treatment. However, on average, patients who developed LR received fewer cycles of high-dose methotrexate (6.7 cycles) than those who did not (9.1 cycles), though this did not reach statistical significance (*p* = 0.092). The occurrence of a deviation in chemotherapy delivery and the time to initiation of adjuvant chemotherapy following surgical resection were not significantly different between those who did or did not develop LR; however, a higher percentage of patients who developed LR resumed chemotherapy >28 days after surgical resection (Table 3). Of the seven patients who developed LR and demonstrated excellent response (≥90% necrosis) to neoadjuvant chemotherapy, three had chondroblastic osteosarcoma. All patients in this group were treated with standard MAP-based regimens consisting of high-dose methotrexate, doxorubicin, and cisplatin.

Primary tumor location, size, grade and surgery type were comparable between those who developed LR and those who did not (Table 2). Notably, one patient with a rotationplasty and one with an upper extremity amputation developed LR. All other cases of LR occurred in patients who had undergone wide resection/limb salvage. Overall, there was no significant difference in margin status between patients who developed LR and those who did not. Of the 14 patients who developed LR, 11 (78.6%) had negative surgical margins. Further, among the 12 patients who had positive surgical margins, only three (25.0%) developed LR.

**Figure 2:**
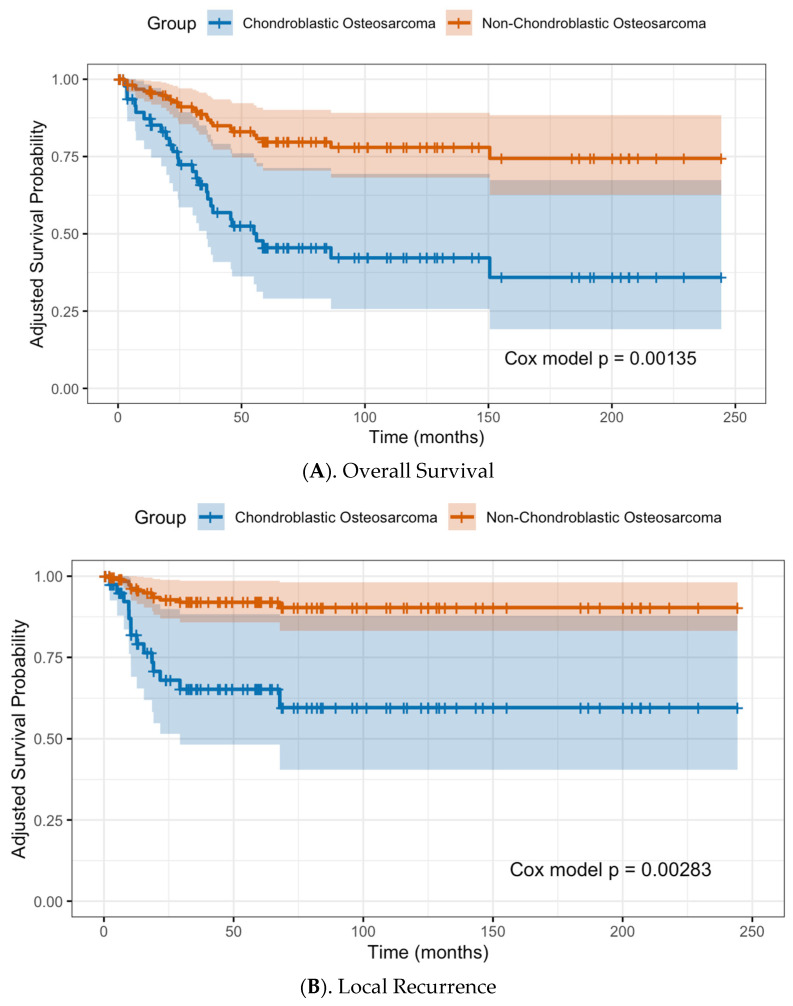
Cox proportional hazards analysis for overall patient survival (**A**) and local recurrence (**B**) in patients with chondroblastic osteosarcoma and those without.

When comparing differences between patient with and without LR, patients who developed LR had a higher incidence of chondroblastic osteosarcoma than those without local recurrence (57.1% vs. 19.3%, *p* < 0.01). The rate of LR in non-chondroblastic osteosarcoma was only 12.2%; while the rate of LR in chondroblastic osteosarcoma was 32.0%. Chondroblastic osteosarcoma was significantly associated with increased odds of local recurrence (OR = 3.74, 95% CI 1.02–13.66, *p* = 0.046), after controlling for tumor location, histological grade, and surgical margins. Patients with chondroblastic osteosarcoma demonstrated a 5.1-fold higher hazard of local recurrence compared with patients with non-chondroblastic osteosarcoma (HR = 5.09; 95% CI 1.75–14.83; *p* = 0.003). Further, patients with chondroblastic osteosarcoma had a 3.46-fold higher hazard of death compared with those with non-chondroblastic subtypes (HR = 3.46; 95% CI 1.62–7.40; *p* = 0.001) (Figure 2).

## 4. Discussion

Osteosarcoma is a rare malignancy with aggressive behavior. The principal objective of surgical intervention in OS is to achieve local control. Studies investigating prognostic factors typically focus on survival and development of metastatic disease. In this study, we evaluated patients with osteosarcoma treated with surgical resection and assessed risk factors for local tumor recurrence. While it is well known that local recurrence correlates with metastatic progression and poor survival, the risks for local recurrence remain poorly understood. It is also unclear whether a locally recurrent tumor itself is the nidus for metastasizing cells, whether local and distant recurrences represent microscopic satellite tumor clusters resistant to chemotherapy, or whether circulating resistant tumor cells are re-seeding the post-surgical site. These open questions highlight the possibility that the biological risk factors for both local recurrence and distal metastasis may overlap.

The 13.7% rate of LR in our total cohort is at the higher end of the reported range [4,7,17,19,20]. For example, one of the largest studies by the Cooperative Osteosarcoma Study Group (COSS) reported a five-year LR rate of only 5.9% [11]. However, in that study, more than 50% of the patients were under the age of 16, and histological subtypes were not reported. Our cohort, treated at a tertiary referral center, reflects a different patient population, with a higher proportion of the aggressive chondroblastic subtype (24.5%) and a higher mean patient age (25.6 years). Given that adult-onset osteosarcoma is associated with less favorable outcomes, these factors likely contribute to the higher LR rate observe in our study and frame our findings as particularly relevant to understanding treatment failure in this higher risk population.

Our single-center retrospective review identified that chondroblastic histologic subtype was the most significant predictor of LR following primary surgical resection. Chondroblastic osteosarcoma remains poorly understood due to its low incidence and variable reporting [21,22]. While the prognostic significance of osteosarcoma subtypes remains a topic of debate, our findings are consistent with large database analyses, such as a recent SEER study of 5950 patients which found that patients with chondroblastic osteosarcoma had significantly worse survival outcomes compared to several other subtypes. Notably, and similar to our findings, patients with chondroblastic osteosarcoma had worse survival outcomes compared to those with telangiectatic, fibroblastic, central, and periosteal osteosarcomas [18]. These findings emphasize the clinical heterogeneity among osteosarcoma subtypes and the need for further consideration of chondroblastic osteosarcoma as a distinct clinical entity in terms of prognostication and requiring specific therapeutic consideration. It is plausible that for tumors with an intrinsically high metastatic and proliferative potential, such as the chondroblastic subtype reported here, the systemic biological drive for recurrence can overcome the local control benefits afforded by achieving a negative margin on the bulk tumor.

Emerging evidence suggests that chondroblastic OS cells may be biologically distinct from osteoblastic cells, highlighting enrichment in hypoxia related signaling pathways [23]. Hypoxia is a well-established driver of tumor aggressiveness and resistance to therapy, while poor vascularization can directly impair the delivery of systemic chemotherapeutic agents, creating “sanctuary sites” where malignant cells can survive treatment [24]. This may provide a mechanistic link between chondroblastic histology and its characteristic chemoresistance and propensity for local recurrence. Tumors with a greater initial burden or more aggressive phenotypes may receive intensified therapy and achieve high rates of necrosis, yet remain predisposed to recurrence due to intrinsic biological behavior and local microenvironmental factors. A detailed mechanistic investigation is beyond the scope of the present manuscript. However, future studies should aim to elucidate these biological drivers through comprehensive molecular profiling and preclinical models to identify relevant biomarkers and therapeutic targets. This represents a critical area for future research that may ultimately inform tailored treatment and surveillance strategies for high-risk subtypes such as chondroblastic osteosarcoma.

While specific chemotherapy agents used, doses delivered, and treatment deviations did not show significant associations with LR risk, there was a trend in which patients with LR received fewer cycles of high-dose methotrexate (HD-MTX) than those who did not develop LR. Though this difference was not statistically significant in our cohort, this study was not designed to specifically investigate this effect. Therefore this does deserve further investigation and may provide additional support for LR, similar to distant progression, being driven more by tumor biology and systemic therapy effectiveness than adequacy of surgical margins. Notably, this finding parallels that from the single-center retrospective study from Wippel et al., which identified a trend towards lower metastasis-free survival in patients who received fewer than 7 cycles of HD-MTX [25], and supports numerous studies demonstrating the correlation between dose intensity of MTX and improved outcomes in osteosarcoma [24,26]. Future hypothesis-driven work is needed to explore how the unique features of the chondroblastic TME drive treatment failure and disease progression.

This study has several limitations, including the relatively small sample size of this retrospective cohort, which may have limited the statistical power of our analyses, increasing the risk of false positive or false negative associations.

This limitation is particularly relevant when interpreting the relationship between surgical margin status, chemotherapy and local recurrence. Additionally, although multivariable models were used to adjust for key confounders, residual confounding cannot be excluded, especially in the context of heterogeneous treatment delivery. As a retrospective study, imaging surveillance was not standardized, and differential follow-up intensity may have influenced the detection of local recurrence, particularly in clinically complex or high-risk cases. This introduces the potential for detection bias, which should be considered when interpreting recurrence rates. The small number of local recurrence events (*n* = 14) constrained the power and stability of our multivariable analysis. To mitigate overfitting, we limited the model to a small set of key variables (grade, site, and margin status). However, other important confounders—such as tumor size, age, histologic response, treatment era, and chemotherapy intensity—were not included. The low events-per-variable ratio may introduce bias, and the association between chondroblastic histology and local recurrence should therefore be interpreted with caution and considered exploratory. These findings are intended to inform future hypothesis-driven research. In this study, margin status, as assessed by subspecialty-trained sarcoma pathologists, was not significantly associated with local recurrence. This finding should not be construed as evidence that surgical margins lack clinical importance, but rather reflects that, within our institutional cohort, documented margin status did not correlate with recurrence outcomes. This observation may be attributable to inherent limitations in histopathologic margin evaluation, the potential for neoadjuvant chemotherapy to eradicate microscopic residual disease beyond the threshold of detection, or underlying tumor biology—such as early local dissemination or hematogenous reseeding of the surgical bed. While these hypotheses are biologically plausible, our conclusions are limited to observed correlations, and further investigation is warranted to elucidate the mechanisms driving local recurrence in osteosarcoma. We acknowledge that the inclusion of low-grade and surface osteosarcoma subtypes introduces heterogeneity, as these variants are typically associated with a lower baseline risk of local recurrence. This may have influenced the observed association between chondroblastic histology and recurrence risk. However, our intent was to evaluate risk factors for local recurrence across the full spectrum of skeletal osteosarcoma encountered in clinical practice, rather than limit the analysis to conventional high-grade cases. We believe this approach provides a more comprehensive assessment of recurrence risk in a real-world tertiary care setting. Importantly, analyses related to chemotherapy delivery were limited to patients with intermediate- or high-grade disease to ensure appropriate clinical relevance and comparability. Differences in medical and surgical management practices across patients may have influenced outcomes in ways not fully captured by our dataset. However, given the rarity of osteosarcoma, conducting the study at a tertiary care center enabled the inclusion of a cohort size comparable to those reported in the existing literature. The cohort was derived from a tertiary referral center, and case mix is therefore subject to referral and selection biases. In particular, the comparatively high proportion of chondroblastic osteosarcoma observed in this series (24.5%) may reflect preferential referral of clinically complex or biologically aggressive disease. Institutional practices, including surgeon experience, operative planning and margin assessment procedures, pathology workflows, and multidisciplinary chemotherapy protocols—may also have influenced both treatment allocation and outcomes in ways not fully measurable in the available data. Heterogeneity in care pathways for patients who received portions of management at outside institutions, as well as variability and incompleteness in chemotherapy records, further introduce potential misclassification and residual confounding. Accordingly, the findings should be interpreted as most applicable to similar tertiary oncology settings and not assumed to generalize to broader osteosarcoma populations managed in community practice. Another limitation is the variability and record completeness in treatment protocols and patient management, particularly for individuals who received portions of their care at outside institutions. This heterogeneity may have influenced the outcomes and introduced confounding factors into the analysis. Future prospective multicenter trials are essential to address these limitations. Such studies would enable the inclusion of larger, more diverse patient populations and facilitate multivariable analyses to better understand how factors such as tumor biology, chemotherapy delivery, and institutional practices influence outcomes. Emerging technologies such as artificial intelligence (AI) and radiomics also offer promising tools for risk stratification. Although most work has focused on soft tissue sarcomas, AI-based radiomic models have shown the ability to predict histologic subtype and recurrence risk from preoperative imaging [27]. Applying these methods to osteosarcoma may allow earlier, noninvasive identification of high-risk subtypes like chondroblastic osteosarcoma, enabling more tailored treatment and surveillance strategies.

## 5. Conclusions

Our findings suggest that while achieving negative margins remains a critical surgical objective in the treatment of osteosarcoma, the intrinsic biology of the chondroblastic subtype may be a more dominant predictor of local failure in this high-risk population. This highlights the potential limitations of relying solely on surgical margin status for assessing the risk of local recurrence, particularly in the modern era of neoadjuvant therapy. The data underscore an urgent need to shift our focus toward biology-driven therapeutic strategies, including the development of novel systemic agents capable of overcoming the inherent chemoresistance and aggressive phenotype of chondroblastic osteosarcoma, to truly improve local control and overall survival for these patients.

## Figures and Tables

**Table 1:** Comparison of Patient Characteristics Between Patients with and without Local Recurrence.

	Total (n = 102)	No LR (n = 88)	LR (n = 14)	*p*-Value
Age, Mean ([min-max]; SD)	25.6 ([4–84]; 16.9)	24.7 ([4–84]; 16.5)	31.3 ([12–67]; 18.9)	0.24
BMI (kg/m^2^), Mean (SD)	25.2 (8.4)	25.0 (8.3)	26.8 (8.8)	0.54
Sex, n (%)				>0.99
Female	46 (45.1%)	40 (45.5%)	6 (42.9%)	
Male	56 (54.9%)	48 (54.5%)	8 (57.1%)	
History of Tobacco Use, n (%)	22 (21.6%)	17 (19.3%)	5 (35.7%)	0.18

LR, local recurrence; n, Number; SD, standard deviation.

**Table 2:** Comparison of Tumor and Surgical Characteristics Between Patients with and without Local Recurrence.

	Total (n = 102)	No LR (n = 88)	LR (n = 14)	*p*-Value
Tumor Size, Mean (SD)	11.0 (5.2)	11.1 (5.37)	10.5 (4.1)	0.65
Tumor Grade, n (%)				0.75
1	8 (7.8%)	8 (9.1%)	0 (0.0%)	
2	13 (12.7%)	11 (12.5%)	2 (14.3%)	
3	73 (71.6%)	62 (70.5%)	11 (78.6%)	
Pelvic Location, n (%)	4 (3.9%)	2 (2.3%)	2 (14.3%)	0.09
Biopsy at OSH, n (%)	15 (14.7%)	13 (14.8%)	2 (14.3%)	>0.99
Amputation, n (%)	10 (9.8%)	9 (10.2%)	1 (7.1%)	0.69
Chondroblastic Osteosarcoma, n (%)	25 (24.5%)	17 (19.3%)	8 (57.1%)	0.005
Positive Surgical Margin, n (%)	12 (11.8%)	9 (10.2%)	3 (21.4%)	0.37
Surgical Margin < 0.1 cm *	14 (13.7%)	13 (14.8%)	1 (7.1%)	0.68
Surgical Margin < 1.0 cm *	61 (59.8%)	51 (58.0%)	10 (71.4%)	0.68
Surgical Margin > 2 cm *	9 (8.8%)	8 (9.1%)	1 (7.1%)	>0.99
Surgery at OSH, n (%)	13 (12.7%)	11 (12.5%)	2 (14.3%)	>0.99

* Closest margin distance in patient with negative margin status; LR, local recurrence; n, Number; OSH, Outside hospital; SD, standard deviation.

**Table 3:** Comparison of Systemic Chemotherapy Between Patients with and without Local Recurrence.

	Total (n = 80)	No LR (n = 69)	LR (n = 11)	*p*-Value
Neoadjuvant, (n, %)	69 (86.3%)	60 (87.0%)	9 (81.8%)	0.43
Treatment Response, (n, %)				0.25
<50%	10 (12.5%)	8 (11.6%)	2 (18.2%)	0.60
[50%, 75%)	15 (18.8%)	14 (20.3%)	1 (9.1%)	0.19
[75%, 90%)	8 (10.0%)	7 (10.1%)	1 (9.1%)	>0.99
≥90%	34 (42.5%)	27 (39.1%)	7 (63.6%)	0.48
Doxorubicin, (n, %)	74 (92.5%)	64 (92.8%)	10 (90.9%)	>0.99
Doxorubicin Cumulative Dose (mg/m^2^), Mean (SD)	397.6 (98.8)	403.7 (91.7)	360.9 (135.9)	0.28
Cisplatin, (n, %)	74 (92.5%)	64 (92.8%)	10 (90.9%)	>0.99
Cisplatin Cumulative Dose (mg/m^2^), Mean (SD)	461.6 (112.6)	471.2 (107.0)	411.3 (135.2)	0.19
Methotrexate, (n, %)	60 (75.0%)	51 (73.9%)	9 (81.8%)	0.68
Number of Cycles of Methotrexate, Mean (SD)	8.5 (3.8)	9.1 (3.7)	6.7 (4.7)	0.092
Ifosfamide, (n, %)	16 (20%)	14 (20.3%)	2 (18.2%)	>0.99
Etoposide, (n, %)	14 (17.5%)	12 (17.4%)	2 (18.2%)	>0.99
Treatment Disruption/Delay, (n, %)	51 (63.8%)	44 (63.8%)	7 (63.6%)	>0.99
Duration to Start of Postoperative Chemotherapy, (n, %), Days				
<14 days	5 (6.3%)	5 (7.2%)	0 (0.0%)	>0.99
[14, 28 days)	30 (37.5%)	26 (37.7%)	4 (36.6%)	0.71
≥28 days	24 (30.3%)	20 (29.0.%)	4 (36.6%)	0.70

## Data Availability

The data presented in this study are available on request from the corresponding author due to ethical protection of patient data and safety.

## Abbreviations

The following abbreviations are used in this manuscript:

BMI	Body mass index
LR	Local recurrence
n	Number
SD	Standard deviation
OSH	Outside Hospital

## References

[1] Mirabello L., Troisi R.J., Savage S.A. (2009). International Osteosarcoma Incidence Patterns in Children and Adolescents, Middle Ages and Elderly Persons. Int. J. Cancer.

[2] Lewis J.J., Leung D., Heslin M., Woodruff J.M., Brennan M.F. (1997). Association of Local Recurrence with Subsequent Survival in Extremity Soft Tissue Sarcoma. J. Clin. Oncol..

[3] Bacci G., Longhi A., Cesari M., Versari M., Bertoni F. (2006). Influence of Local Recurrence on Survival in Patients with Extremity Osteosarcoma Treated with Neoadjuvant Chemotherapy: The Experience of a Single Institution with 44 Patients. Cancer.

[4] Weeden S., Grimer R.J., Cannon S.R., Taminiau A.H., Uscinska B.M., Intergroup European Osteosarcoma (2001). The Effect of Local Recurrence on Survival in Resected Osteosarcoma. Eur. J. Cancer.

[5] Takeuchi A., Lewis V.O., Satcher R.L., Moon B.S., Lin P.P. (2014). What Are the Factors That Affect Survival and Relapse after Local Recurrence of Osteosarcoma?. Clin. Orthop. Relat. Res..

[6] Rodriguez-Galindo C., Shah N., McCarville M.B., Billups C.A., Neel M.N., Rao B.N., Daw N.C. (2004). Outcome after Local Recurrence of Osteosarcoma: The St. Jude Children’s Research Hospital Experience (1970–2000). Cancer.

[7] Bertrand T.E., Cruz A., Binitie O., Cheong D., Letson G.D. (2016). Do Surgical Margins Affect Local Recurrence and Survival in Extremity, Nonmetastatic, High-Grade Osteosarcoma?. Clin. Orthop. Relat. Res..

[8] Nathan S.S., Gorlick R., Bukata S., Chou A., Morris C.D., Boland P.J., Huvos A.G., Meyers P.A., Healey J.H. (2006). Treatment Algorithm for Locally Recurrent Osteosarcoma Based on Local Disease-Free Interval and the Presence of Lung Metastasis. Cancer.

[9] He F., Zhang W., Shen Y., Yu P., Bao Q., Wen J., Hu C., Qiu S. (2016). Effects of Resection Margins on Local Recurrence of Osteosarcoma in Extremity and Pelvis: Systematic Review and Meta-Analysis. Int. J. Surg..

[10] Loh A.H., Navid F., Wang C., Bahrami A., Wu J., Neel M.D., Rao B.N. (2014). Management of Local Recurrence of Pediatric Osteosarcoma Following Limb-Sparing Surgery. Ann. Surg. Oncol..

[11] Andreou D., Bielack S.S., Carrle D., Kevric M., Kotz R., Winkelmann W., Jundt G., Werner M., Fehlberg S., Kager L. (2011). The Influence of Tumor- and Treatment-Related Factors on the Development of Local Recurrence in Osteosarcoma after Adequate Surgery. An Analysis of 1355 Patients Treated on Neoadjuvant Cooperative Osteosarcoma Study Group Protocols. Ann. Oncol..

[12] Matsuo T., Sugita T., Sato K., Hotta T., Tsuchiya H., Shimose S., Kubo T., Ochi M. (2005). Clinical Outcomes of 54 Pelvic Osteosarcomas Registered by Japanese Musculoskeletal Oncology Group. Oncology.

[13] Papakonstantinou E., Stamatopoulos A., Athanasiadis D.I., Kenanidis E., Potoupnis M., Haidich A.B., Tsiridis E. (2020). Limb-Salvage Surgery Offers Better Five-Year Survival Rate Than Amputation in Patients with Limb Osteosarcoma Treated with Neoadjuvant Chemotherapy. A Systematic Review and Meta-Analysis. J. Bone Oncol..

[14] Poudel R.R., Tiwari V., Kumar V.S., Bakhshi S., Gamanagatti S., Khan S.A., Rastogi S. (2017). Factors Associated with Local Recurrence in Operated Osteosarcomas: A Retrospective Evaluation of 95 Cases from a Tertiary Care Center in a Resource Challenged Environment. J. Surg. Oncol..

[15] Bacci G., Forni C., Longhi A., Ferrari S., Mercuri M., Bertoni F., Serra M., Briccoli A., Balladelli A., Picci P. (2007). Local Recurrence and Local Control of Non-Metastatic Osteosarcoma of the Extremities: A 27-Year Experience in a Single Institution. J. Surg. Oncol..

[16] Donati D., Giacomini S., Gozzi E., Ferrari S., Sangiorgi L., Tienghi A., DeGroot H., Bertoni F., Bacchini P., Bacci G. (2004). Osteosarcoma of the Pelvis. Eur. J. Surg. Oncol..

[17] Brosjo O. (1999). Surgical Procedure and Local Recurrence in 223 Patients Treated 1982–1997 According to Two Osteosarcoma Chemotherapy Protocols. The Scandinavian Sarcoma Group Experience. Acta. Orthop. Scand..

[18] Sun H.H., Chen X.Y., Cui J.Q., Zhou Z.M., Guo K.J. (2018). Prognostic Factors to Survival of Patients with Chondroblastic Osteosarcoma. Medicine.

[19] Bacci G., Ferrari S., Mercuri M., Bertoni F., Picci P., Manfrini M., Gasbarrini A., Forni C., Cesari M., Campanacci M. (1998). Predictive Factors for Local Recurrence in Osteosarcoma: 540 Patients with Extremity Tumors Followed for Minimum 2.5 Years after Neoadjuvant Chemotherapy. Acta Orthop. Scand..

[20] Bielack S.S., Kempf-Bielack B., Delling G., Exner G.U., Flege S., Helmke K., Kotz R., Salzer-Kuntschik M., Werner M., Winkelmann W. (2002). Prognostic Factors in High-Grade Osteosarcoma of the Extremities or Trunk: An Analysis of 1702 Patients Treated on Neoadjuvant Cooperative Osteosarcoma Study Group Protocols. J. Clin. Oncol..

[21] Junior A.T., de Abreu Alves F., Pinto C.A., Carvalho A.L., Kowalski L.P., Lopes M.A. (2003). Clinicopathological and Immunohistochemical Analysis of Twenty-Five Head and Neck Osteosarcomas. Oral. Oncol..

[22] Bacci G., Bertoni F., Longhi A., Ferrari S., Forni C., Biagini R., Bacchini P., Donati D., Manfrini M., Bernini G. (2003). Neoadjuvant Chemotherapy for High-Grade Central Osteosarcoma of the Extremity. Histologic Response to Preoperative Chemotherapy Correlates with Histologic Subtype of the Tumor. Cancer.

[23] Zheng X., Wu W., Zhao Z., Zhang X., Yu S. (2024). Single-Cell Transcriptomic Insights into Chemotherapy-Induced Remodeling of the Osteosarcoma Tumor Microenvironment. J. Cancer Res. Clin. Oncol..

[24] Ferrari S., Bacci G., Picci P., Mercuri M., Briccoli A., Pinto D., Gasbarrini A., Tienghi A., del Prever A.B. (1997). Long-Term Follow-up and Post-Relapse Survival in Patients with Non-Metastatic Osteosarcoma of the Extremity Treated with Neoadjuvant Chemotherapy. Ann. Oncol..

[25] Wippel B., Gundle K.R., Dang T., Paxton J., Bubalo J., Stork L., Fu R., Ryan C.W., Davis L.E. (2019). Safety and Efficacy of High-Dose Methotrexate for Osteosarcoma in Adolescents Compared with Young Adults. Cancer Med..

[26] Delepine N., Delepine G., Bacci G., Rosen G., Desbois J.C. (1996). Influence of Methotrexate Dose Intensity on Outcome of Patients with High Grade Osteogenic Osteosarcoma. Analysis of the Literature. Cancer.

[27] Schmitz F., Voigtlander H., Jang H., Schlemmer H.P., Kauczor H.U., Sedaghat S. (2024). Predicting the Malignancy Grade of Soft Tissue Sarcomas on Mri Using Conventional Image Reading and Radiomics. Diagnostics.

